# Channel-length dependence of particle diffusivity in confinement†

**DOI:** 10.1039/d1sm00289a

**Published:** 2021-04-06

**Authors:** Soichiro Tottori, Karolis Misiunas, Vahe Tshitoyan, Ulrich F. Keyser

**Affiliations:** Cavendish Laboratory, Department of Physics, University of Cambridge CB3 0HE UK ufk20@cam.ac.uk

## Abstract

Understanding the diffusive behavior of particles and large molecules in channels is of fundamental importance in biological and synthetic systems, such as channel proteins, nanopores, and nanofluidics. Although theoretical and numerical modelings have suggested some solutions, these models have not been fully supported with direct experimental measurements. Here, we demonstrate that experimental diffusion coefficients of particles in finite open-ended channels are always higher than the prediction based on the conventional theoretical model of infinitely long channels. By combining microfluidic experiments, numerical simulations, and analytical modeling, we show that diffusion coefficients are dependent not only on the radius ratio but also on the channel length, the boundary conditions of the neighboring reservoirs, and the compressibility of the medium.

## Abstract 2

The diffusion coefficients of particles in finite open-ended channels are always higher than the prediction based on the conventional theoretical model of infinitely long channels when the flow is permitted through the channels.

## Graphical abstract

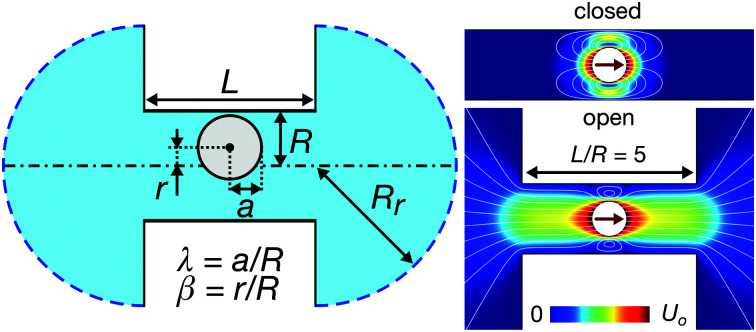

## 1 Introduction

Diffusion plays an important role in confined spaces due to the suppression of advection, for example, in biological tissues (such as kidneys^1^ and brains^2^), gels,^3^ and separation membranes.^4,5^ In particular, channels are of great interest due to their relevance to biological and synthetic transport systems, including membrane proteins, nanofluidic devices, and nanopores. In recent years, towards the aim of enhancing the sensitivity in nanopore sensing as well as the energy efficiency in filtration processes, thinner membranes (*i.e.*, short channels) have been attracting attention.^6–10^ Of special interest is the analysis of proteins including their size and shape extracted from complex signals in nanopores.^7,9,11–14^

Historically, diffusion of large molecules and particles in channels has been investigated indirectly, relying on the model-based extraction of particle diffusivity.^15,16^ However, these bulk measurements of large ensembles of particles do not allow detailed analysis of individual particle behavior. Direct tracking of particle motions in channels has become possible only recently by the combination of micro-fabricated optically transparent channels, high-resolution optical microscopy, and fast digital cameras.^8,17,18^ Here, by utilizing these technologies, we investigated particle diffusion in finite-length channels to elucidate the effects of channel length and open/closed reservoir boundary conditions on the diffusion coefficients in channels. Such measurements of both passive diffusion and driven transport allow for highly controlled investigations of the position- and channel-dependent characteristics at the single particle level.

We first show that experimentally measured diffusion coefficients in finite channels are always larger than the classical theoretical prediction of diffusivity in an infinitely long channel. Second, we investigate the channel-length dependency of diffusion coefficients by experimental measurements, finite element simulations, and analytical modeling. Finally, we examine the influence of the outer boundary conditions of reservoirs by calculating the critical reservoir size above which the net flow across a channel is absorbed due to the finite compressibility of water.

## 2 Materials and methods

**Fig. 1:**
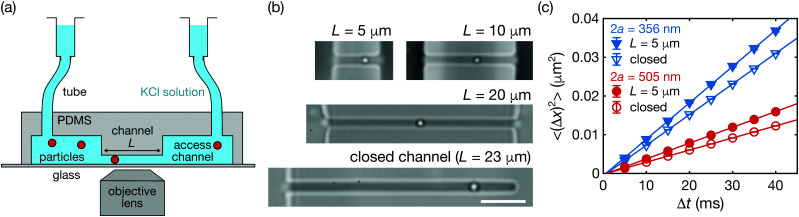
Measurement of diffusion coefficients in channels. (a) Schematic illustration of our experimental system. (b) Microfluidic channels with various channel lengths *L* and boundary conditions. Scale bar equals 5 μm. (c) Mean square displacement (MSD) of particles inside the open (*L* = 5 μm) and the closed channels. Solid lines are linear fits to the measured points. Error bars (hidden within symbols) denote the standard errors.

Experiments were performed using our experimental system consisting of an optical microscope and a microfluidic chip, as shown in Fig. 1(a).^19,20^ Images were recorded with a CMOS camera (MC1362, Mikrotron) *via* a 100×, NA 1.4 objective lens (UPLSAPO, Olympus) at 200 frames per second. Fig. 1(b) shows the microfluidic channels with lengths between *L* = 5 μm and *L* = 28 μm. The channels were fabricated by a standard soft lithography technique using polydimethylsiloxane (PDMS, Sylgard 184, Dow) bonding onto glass cover slips. One or both of the channel ends are open to the access channels with a height of 10 μm. Crucially, we ensure that the cross-section of the channel remains the same for all chips to maintain constant radius ratios. Hence, we fabricated a robust master mold from a silicon wafer with rectangular structures of length 75 μm, height and width 0.75 ± 0.05 μm, using electron-beam lithography and reactive ion Si etching (see ESI,† for more detail). The molds for the access channels were subsequently deposited on top of the silicon rectangular structures *via* photolithography (AZ 9260, AZ Electronic Materials GmbH). The silicon master mold was reused by removing the access channels and PDMS residuals^21^ and subsequently redepositing new photoresists with different gaps. We used polystyrene particles (Polybead®, Polysciences Inc.) of two different diameters (2a = 356 ± 14, 505 ± 8 nm) dispersed stably in a 5 mM KCl solution due to their negatively charged surfaces.^20^

## 3 Results and discussions

Fig. 1(c) shows the typical mean square displacements (MSD) of single particles (2*a* = 356, 505 nm) inside the closed or open channel (*L* = 5 μm) along the channel axis (the *x*-axis). For each data set, the linear function

1 〈(Δ*x*)^2^〉 = 2*D*_*x*_Δ*t* + const.

was fitted to the measured points to calculate the diffusion coefficients *D*_*x*_.

**Fig. 2:**
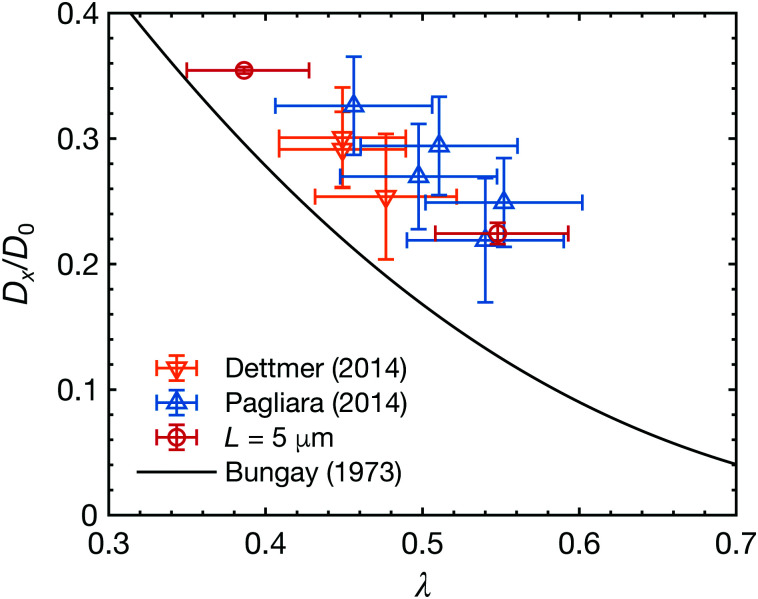
Normalized diffusion coefficients of particles inside finite-length open channels as a function of the radius ratio *λ* (= *a*/*R*). Data points are from the previous works by Dettmer *et al.*^17^ (▽) and Pagliara *et al.*^22^ (△) (2*a* = 505 μm, *L* = 5 μm), and our measurements in this work (2*a* = 356, 505 nm, *L* = 5 μm) (○). The solid line is the analytical expression for a concentric sphere in an infinitely long channel obtained by Bungay and Brenner.^23^

In Fig. 2, we plot the diffusion coefficients of particles inside finite-length open channels (*L* = 5 μm) with various cross-sections as a function of the radius ratio, *λ*, where *λ* is the ratio of particle radius to channel radius, *λ* = *a*/*R*. Each diffusion coefficient is normalized with its bulk value,2
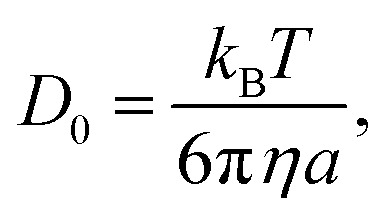
where *k*_B_, *T*, and *η* are the Boltzmann constant, temperature, and viscosity of water, respectively. The data were obtained from the measurements in this work as well as from literature.^17,22^ The radius, *R*, was approximated with a circle of an equal cross-sectional area^17,22^ or estimated by mapping the experimental and theoretical results of closed channels (see ESI† for more detail). The solid line is the analytical expression derived by Bungay and Brenner^23^ for a concentric sphere in an infinitely long channel. Notably, the experimental results are always higher than those of the theoretical prediction, implying that the boundary conditions of the channel ends have a significant influence on the diffusive behavior of particles in channels.

**Fig. 3:**
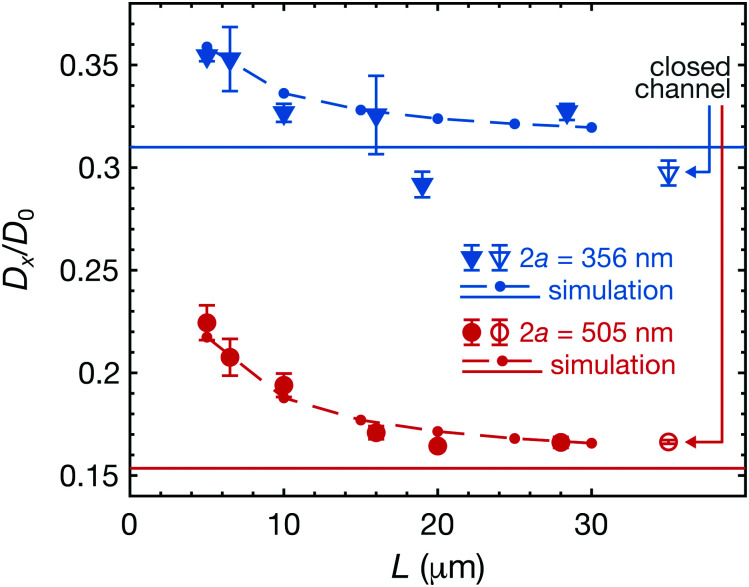
Normalized averaged diffusion coefficients 〈*D*_*x*_〉/*D*_0_ as a function of the channel length. The filled/unfilled markers indicate experimental measurements, where error bars denote the standard errors. The rightmost unfilled markers are the measurements in the closed channel (*L* = 23 μm). The dots and solid lines show the simulation results of the open channels and the closed channels, respectively (the dashed lines are guides to the eye). The radius ratios are *λ* ≈ 0.386 and 0.548 for 2*a* = 356 and 505 nm, respectively.

To investigate the effects of the boundary conditions of the channel ends, we measured diffusion coefficients of polystyrene particles in various channel lengths from 5 μm to 28 μm. In Fig. 3, we show the normalized diffusion coefficients of particles with two different diameters (505 nm and 356 nm) as a function of the channel length. The rightmost unfilled markers indicate the measurements in the closed channels (*L* = 23 μm). Our measurements showed that the diffusion coefficients increase for shorter open channels for both sizes.

**Fig. 4:**
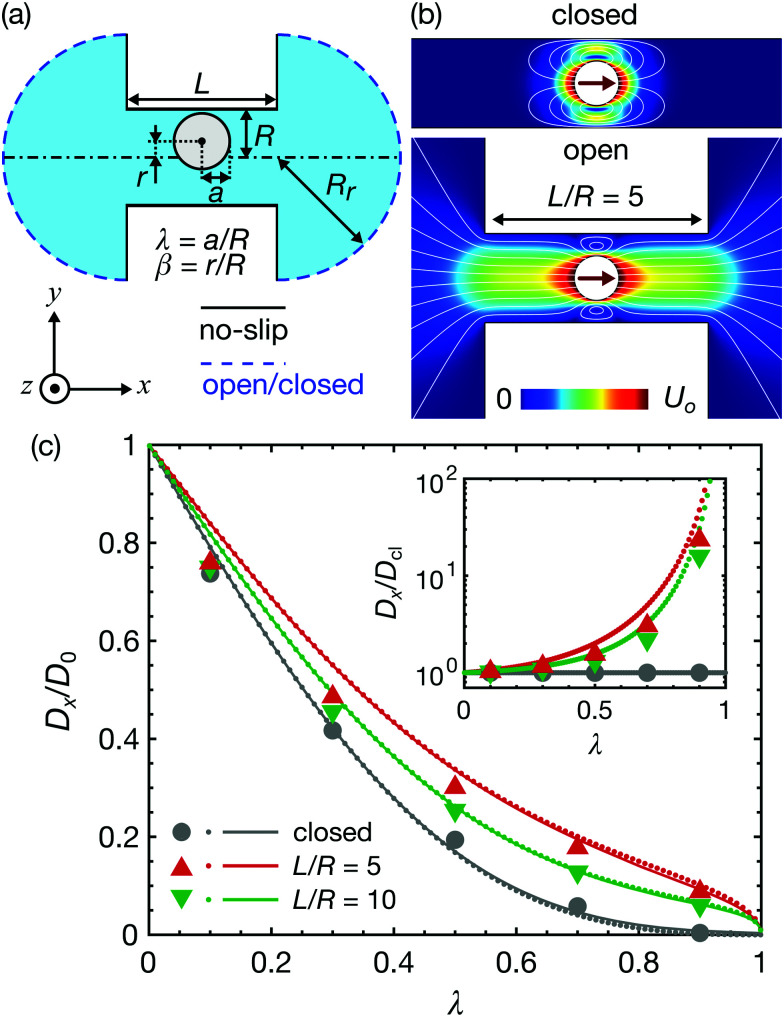
Finite element simulations and analytical models. (a) Simulation domain and boundary conditions. (b) Simulated flow profile in a closed channel and a finite open channel (*λ* = 0.5, *L*/*R* = 5). (c) Simulated normalized diffusion coefficients as a function of the radius ratio. Markers indicate the simulated average diffusion coefficients, 〈*D*_*x*_〉/*D*_0_. Dots and solid lines are the results of the simulation and analytical models of concentric spheres (*β* = 0), respectively. (Inset) Simulated diffusion coefficients normalized with those of closed channels, *D*_*x*_(*β* = 0)/*D*_cl_ (dots), and 〈*D*_*x*_〉/〈*D*_cl_〉 (markers).

To understand the changes of diffusion coefficients, numerical simulations of hydrodynamic drag on a sphere inside a channel were performed using a finite element method. The Stokes equations (∇*p* = *μ*∇^2^***u***,∇·***u*** = 0, where *p* and ***u*** are the pressure and the velocity of fluid) were solved using a finite element method (COMSOL Multiphysics V4.4) for a cylindrical channel with both ends connected to sufficiently large reservoirs, as shown in Fig. 4(a). All solid surfaces, including the channel and particle, have no-slip boundary conditions, and the ends of the reservoirs are assigned either open or no-slip boundary conditions.

The local diffusion coefficients *D*_*x*_(*β*), where *β* is the dimensionless radial position, *β* = *r*/*R*, were then calculated using the simulated hydrodynamic coefficients (see ESI† for more details). The averaged diffusion coefficients along a channel axis 〈*D*_*x*_〉 can be calculated by integrating local diffusion coefficients *D*_*x*_(*β*) along the radial direction as^4^3
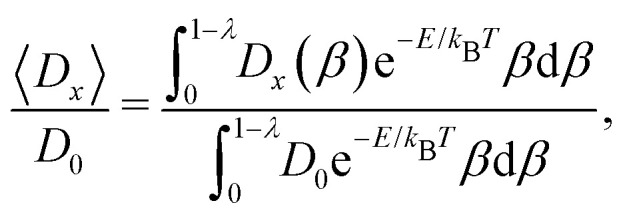
where *E* is a potential (*e.g.*, electrostatic). We set *E* = 0 for the following calculations as the Debye length is small enough (≈4 nm) in our experiments.

Using eqn (3), the simulated diffusion coefficients in open and closed channels are plotted with dots and solid lines in Fig. 3, respectively (the dashed lines are guides to the eye). For both cases, we observed good agreement between the experiments and simulations. To find the best approximation of an effective radius for our square channel in the experiments, here we simulated diffusion coefficients of closed cylindrical channels and then fitted the results to the experimentally obtained data of the closed channels. By using the least squares method, we obtained 2*R* ≈ 922 nm, yielding the radius ratios *λ* ≈ 0.386 and 0.548 for 2a = 356 and 505 nm, respectively (see ESI† for more detail).

The underlying mechanism of the variation in diffusion coefficients with channel lengths can be clearly observed in the flow profiles around particles, as shown in Fig. 4(b). In a closed channel, all flow circulates around a particle, as no net flow is permitted. In contrast, in a finite open channel, net flow is partially permitted through the channel by the piston-like mechanism. This additional flow mode significantly reduces the hydrodynamic friction of the particles, thus increasing the particle diffusion coefficients. Previously, it was reported that this net flow throughout the entire channel induced non-decaying interactions between multiple particles.^24^

In Fig. 4(c), the simulated normalized diffusion coefficients as a function of the radius ratio are summarized. The markers and dots indicate the simulated average values 〈*D*_*x*_〉/*D*_0_ and the center-line values *D*_*x*_(*β* = 0)/*D*_0_, respectively. The average values were calculated by taking the average of the local diffusion coefficients of the 10 divided segments (see ESI† for detailed simulation results). For a closed channel, the averaged diffusion coefficients are below the center-line values for small *λ*, but exceed the center-line value at *λ* ≳ 0.3. In contrast, the averaged diffusion coefficients are always smaller than the center-line values for relatively short open-ended channels (*e.g.*, *L*/*R* = 5,10). The inset of Fig. 4(c) shows the diffusion coefficients normalized with those of the corresponding closed channels. The difference in diffusion coefficients between open and closed channels can be larger than one order of magnitude for *λ* approaching unity.

The solid lines in Fig. 4(c) are the results of the analytical model. Due to the linearity of Stokes’ equations, the force *F*, torque *T*_o_, and additional pressure drop force due to the existence of a particle in an infinitely long channel Δ*p*^+^*A*, where *A* is the cross-sectional area of the channel *A* = π*R*^2^, can be described as linear functions of the characteristic particle velocities *U*_o_ and *aΩ* as well as the mean flow velocity in a channel *u*_m_. Bungay and Brenner described this relation using the following matrix equation,^23^4
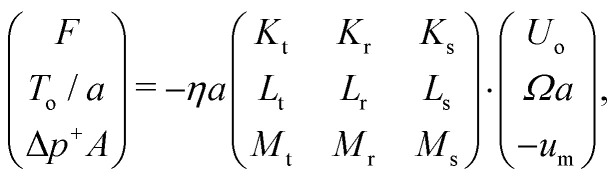
where the matrix elements *K*, *L*, and *M* are dimensionless constants. The total pressure drop between two reservoir ends Δ*p* can be described as

5 Δ*p* = Δ*p*^+^ + Δ*p*_c_,

where Δ*p*_c_ is the pressure drop across an empty channel, which can be approximated by the sum of the Sampson entrance resistance and Poiseuille flow resistance as^25,26^6
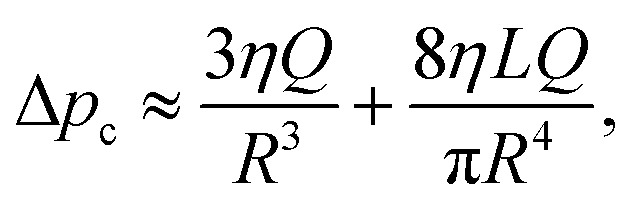
where *Q* is the flow rate, *Q* = *Au*_m_. Thus, for a concentric sphere, the hydrodynamic friction coefficients *ξ* (= *F*/*U*_o_) for an open (Δ*p* = 0) and a closed channel (*u*_m_ = 0) can be calculated as7
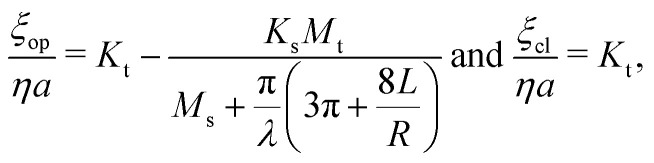
respectively. The corresponding normalized diffusion coefficients are *D*_op(cl)_/*D*_0_ = 6π*ηa*/*ξ*_op(cl)_. For the elements *K* and *M*, we used the expressions derived by Bungay^23^ summarized in ESI.† The results show excellent agreement with those of simulations of a concentric sphere.

When one of the reservoirs' ends is closed, the net flow across a channel always becomes zero, regardless of the size of the reservoirs, as we assume steady state conditions with an incompressible fluid (*ρ* = const.) in the analysis so far. In reality, however, the compressibility of water is small but does exist (≈4.6 × 10^−10^ Pa^−1^, at 20 °C under the atmospheric pressure^27^). With the channel-reservoir geometrical configuration shown here, even a minute variation in the volume of water inside the reservoirs can be amplified and affect the flow inside a channel. In other words, the net flow across a channel can be absorbed into closed reservoirs.

To analyze the effect of compressibility of the medium, first, we describe the compressibility of water by using the empirical model of isothermal compressibility of water proposed by Fine and Millero.^27^ The specific volume of water *V*(*p*,*T*) (the inverse of density, *ρ*^−1^) for a small gauge pressure *p* is modeled empirically as8
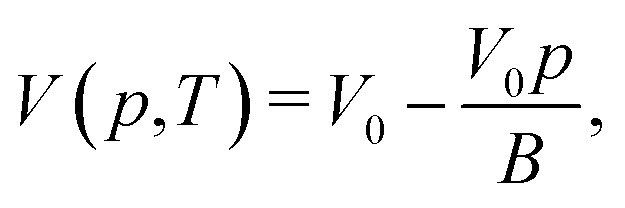
where *B*^−1^ and *V*_0_ are the compressibility of water and the specific volume at *p* = 0 (*i.e.*, 1 atm), respectively. Note that *p* is defined as a gauge pressure in pascals. By defining the averaged displacement of water flow in a channel *x*_m_ as

9 *ẋ*_m_ = *u*_m_,

the total pressure drop force on the cross-section of the channel can be expressed as

**Fig. 5:**
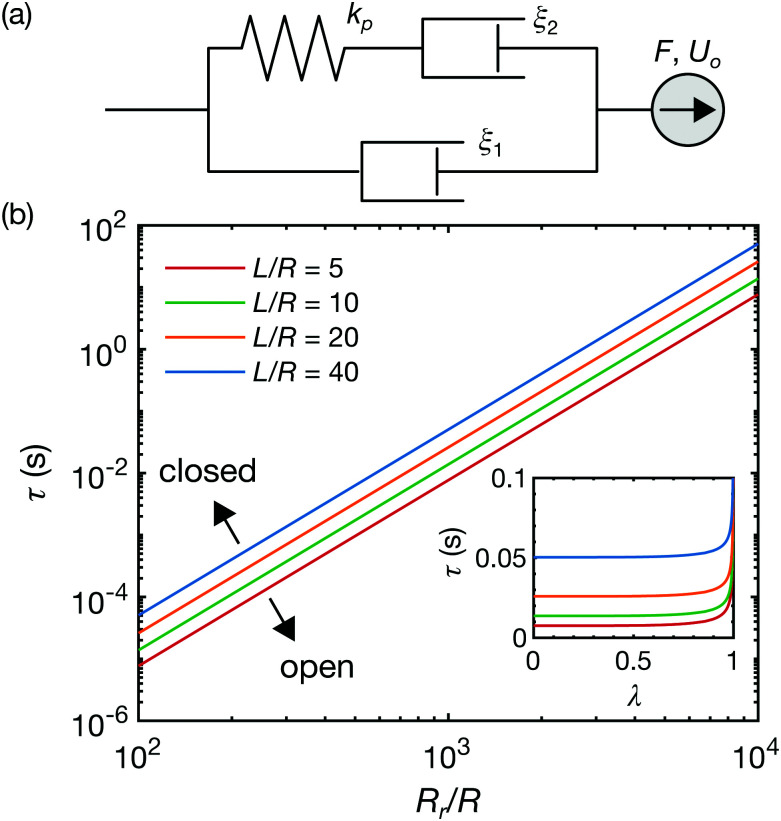
Viscoelastic model of a particle inside a channel connected with closed reservoirs. (a) Schematic representation of a viscoelastic Jeffreys fluid model (eqn (13)). (b) Time constant *τ* as a function of the dimensionless reservoir radius. The radius ratio is fixed at *λ* = 0.5. The system works as an open or a closed channels below or above the slopes, respectively. (Inset) Time constants *τ* as a function of the radius ratio. The reservoir radius is fixed at *R*_r_/*R* = 10^3^.

10 Δ*pA* = −*k*_c_*x*_m_,

where *k*_c_ is the spring constant obtained by considering the water in the reservoirs as a spring with a spring constant,11
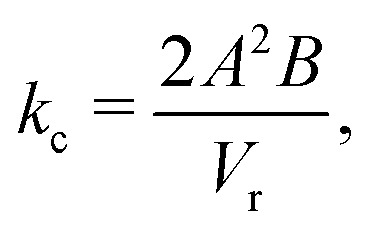
where *V*_r_ is the volume of one reservoir, and the coefficient ‘2’ indicates that there are closed reservoirs at both ends of the channel. The volume of one reservoir *V*_r_ is calculated as a hemisphere with radius *R*_L_,12
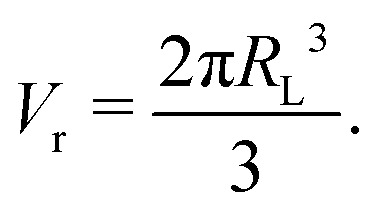
Note that here we neglect the compression/expansion of the water inside the channel. By substituting eqn (10) into eqn (5), we obtain a Jeffreys viscoelastic model (a standard linear fluid model) shown in Fig. 5(a),13
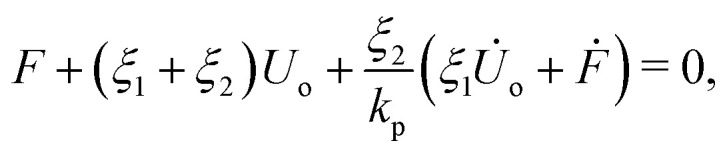
where *ξ*_1_ and *ξ*_2_ are

14 *ξ*_1_ = *ξ*_op_, and *ξ*_2_ = *ξ*_cl_ − *ξ*_op_,

respectively, and *k*_p_ is the effective spring constant for the particle,15
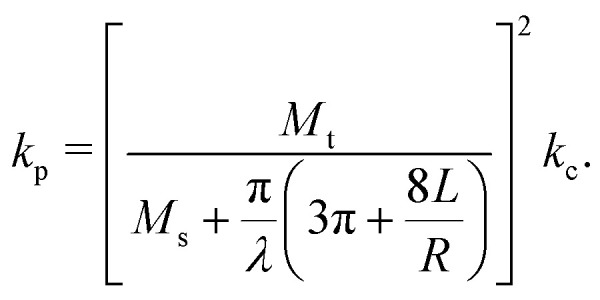
The verification of the model was performed using finite element simulations (see ESI† for more details).

It is known that mean square displacement in such a Jeffreys fluid takes the form of^28^16
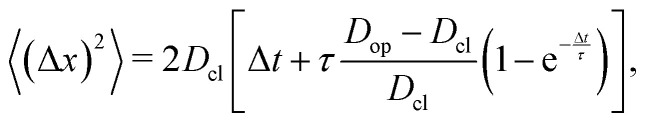
where *τ* is the time constant17
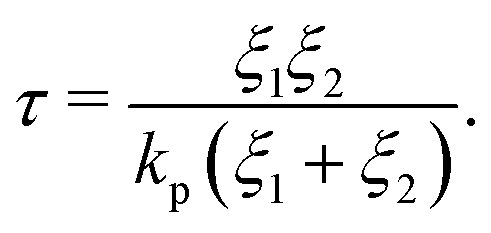
Thus, when reservoirs are large or the lag time is sufficiently short, eqn (16) recovers, *D*(Δ*t*/*τ* → 0) → *D*_op_, whereas in the opposite case, *D*(Δ*t*/*τ* → ∞) → *D*_cl_. In Fig. 5(b), the time constants *τ* of typical geometrical parameters (*λ* = 0.5 and *L*/*R* = 5,10,20,40) are plotted as a function of the reservoir radius *R*_r_/*R*. From eqn (17), *τ* scales as *τ* ∼ (*R*_r_/*R*)^3^ for given constant *λ* and *L*/*R*. The regime above the slopes is where the reservoirs work as closed boundaries, whereas the regime below the slope is where the reservoirs work effectively as open boundaries. For example, assuming the time scale of the particle tracking to be Δ*t* ∼ 1 × 10^−2^ s, the critical reservoir size can be estimated to be roughly *R*_r_/*R* ∼ 1 × 10^−3^. Thus, for a channel with a radius of a 1 μm, a reservoir with a radius below 1 mm works as a closed channel. Conversely, for the reservoir radius larger than 1 mm, the particles diffuse as if they are in a channel connected with open reservoirs because the net flow is absorbed by the reservoirs. The inset shows the time constants as a function of *λ* at the fixed *R*_r_/*R* = 10^3^ for various *L*/*R*. *τ* is found to be relatively insensitive to *λ* and scales with *L*/*R* as *τ* ∼ *L*/*R*.

## 4 Conclusions

In conclusion, we have demonstrated that diffusion coefficients of particles inside finite open channels depend not only on the radius ratios but also on the channel lengths and the boundary conditions. Our microfluidic experiments with various channel lengths and boundary conditions showed variations in diffusion coefficients and were quantitatively in agreement with the results of our numerical simulations and analytical models. Classically, the particle motions in a channel have been studied based on the assumption of no net flow across the channel. Our study, combining experimental, numerical, and analytical studies, shows that for relatively short channels, this assumption can lead to underestimating diffusion coefficients. Furthermore, we quantify the critical length scale of reservoirs that affects the diffusion of particles in channels, by using finite element simulations and analytical modeling. The obtained characteristic time scale provides a criterion for determining the the effects of boundary conditions. Our results are relevant to various diffusion phenomena in biological and synthetic systems, including molecular transport through biological membranes and in intra/extracellular spaces, micro-/nano-fluidic devices, and porous materials, such as gels and rocks.

## Conflicts of interest

There are no conflicts to declare.

## Supplementary Material

SM-017-D1SM00289A-s001
