# Supplementary material for: Channel-length dependence of particle diffusivity in confinement
Source: Soft Matter. 2021 Apr 6;17(20):5131–6. doi: 10.1039/d1sm00289a (PMC8151877; doi:10.1039/d1sm00289a)
Supplement: SM-017-D1SM00289A-s001 [file SM-017-D1SM00289A-s001.pdf]

Electronic Supplementary Material (ESI) for Soft Matter.
This journal is © The Royal Society of Chemistry 2021

Electronic Supplementary Information (ESI): Channel-length dependence of particle diffusivity in confinement

Soichiro Tottori, Karolis Misiunas, Vahe Tshitoyan, and Ulrich F. Keyser*
Cavendish Laboratory, Department of Physics, University of Cambridge, CB3 0HE, United Kingdom
(Dated: March 30, 2021)

CONTENTS

I. Channel fabrication 2
A. Reactive ion etching (RIE) of silicon wafers 2
B. Photolithography 2
C. Removal of residual PDMS 2

II. Simulations of diffusion coefficients 3
A. Hydrodynamic frictions and diffusion coefficients 3
B. Local diffusion coefficient 3
C. Effective radius of the channel 3

III. Analytical modeling of diffusion coefficients 4
A. Analytical expressions of matrix elements in Eq. 2 4
B. Compressibility of water 4
C. Finite element simulation of a particle in a closed system 5

References 6

* ufk20@cam.ac.uk

I. CHANNEL FABRICATION

A. Reactive ion etching (RIE) of silicon wafers

First, a silicon wafer was cleaned with acetone and isopropanol (IPA). Sonication was not used because it occasionally damaged the edges of the sample. Subsequently, oxygen plasma ashing was performed at around 150 W for about a minute. Photoresist PMMA A6 was spin-coated at 1500-2000 rpm to form a layer with a thickness of ∼1 µm. The chip was then baked on a hotplate for 7 min at 180 °C. Using e-beam lithography, the designed patterns were written into the photoresist. Development was performed with MIBK/IPA (25%/75%) + 2% deionized water for 45 s, followed by rinsing in IPA for at least 30 s. After the development, oxygen plasma ashing was performed for 10 s to remove any residual PMMA. Note that this process needed to be finished within 10 s because it may otherwise lead to rounding the patterned edges. The chip was then baked again for about 5 min at 110 °C to remove any adsorbed water. A layer of Cr/Au (25 nm/375 nm) was deposited via thermal evaporation. Other metals can be substituted as long as they are around 400 nm in thickness and adhere well to this substrate (e.g., Ti instead of Cr). Standard acetone lift-off was performed. Ashing for 1 min and baking for 10 min were performed to remove any residuals before loading it into the etcher. The rate of etching is system-specific and needs to be optimised for each different setup. We used CF_4 + $SiCl_4$ combined with argon at 300 W for 7 min, which resulted in approximately 750 nm in height with sharp edges. The effective etching rate of the Cr/Au layer was about half of that of Si. Thus, after 7 min, the whole metal was removed, and only Si wire structures with a height of 750 nm were left. The scanning electron microscopy images of the final fabricated Si structures are shown in Fig. S1.

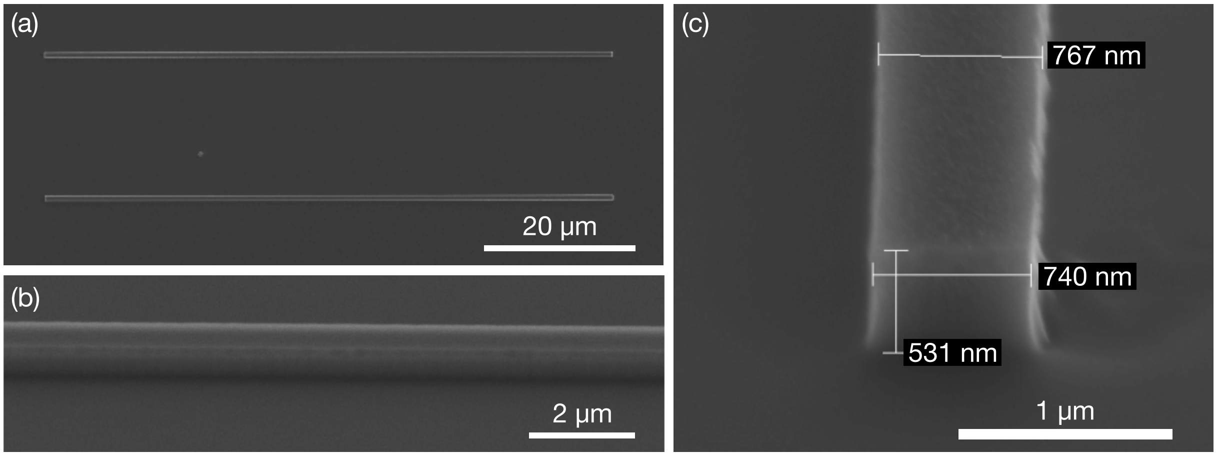

FIG. S1: Si channel molds fabricated with RIE. (a) Overview from the top. (b) Zoomed-up view with a tilt angle of 45°. The channel sides are smooth without defects. (c) Cross-sectional view with a tilt angle of 45°. The height is approximately 751 nm (≈ 531 nm/ sin (45°)).

B. Photolithography

The detailed protocols of photolithography are summarized here. We used a positive photoresist (AZ 9260, AZ Electronic Materials GmbH) to fabricate access channels. The fabrication steps are the following: (i) Spin-coating with a spin speed of 2000 rpm and a ramp of 1000 rpm/s for 30 s. (ii) Bake at 115 °C for 3 min on a contact hotplate. (iii) UV light exposure (365-405 nm, 52 mW/cm^2) with a mask aligner. (iv) Development with a developer (AZ400k, MicroChemicals) diluted with water at a ratio of 4 : 1 for several seconds. The final thickness was approximately 10 µm.

C. Removal of residual PDMS

We found that the surface of the silicon mold used for PDMS replication became contaminated with an invisibly thin layer of PDMS. To remove the PDMS residual from the surface, we cleaned the silicon molds with tetrabutylammonium fluoride (TBAF) [1]. The protocol is summarized in the following. TBAF was diluted by 1% in propylene glycol

methyl ether acetate (PMA). The silicon chip was immersed into this mixture of TBAF and PMA on a hotplate at the temperature of 50°C for 15 min. Subsequently, the silicon chip was immersed into PMA at a temperature of 50°C for 15 min and finally rinsed with IPA. If the surface is not clean enough, the procedure needs to be repeated.

II. SIMULATIONS OF DIFFUSION COEFFICIENTS

A. Hydrodynamic frictions and diffusion coefficients

When a particle is off-centered in a channel, it experiences not only a hydrodynamic drag force but also a torque. In general, in the Stokes flow, the relation between the force $\boldsymbol{F}$ and torque $\boldsymbol{T}$ that a solid body experiences in fluid and its translational velocity $\boldsymbol{U}$ and rotational velocity $\boldsymbol{\Omega}$ is described as [2]

$$\begin{pmatrix} \boldsymbol{F} \\ \boldsymbol{T} \end{pmatrix} = -\begin{pmatrix} \boldsymbol{\xi}_T & \boldsymbol{\xi}_{TR} \\ \boldsymbol{\xi}_{RT} & \boldsymbol{\xi}_R \end{pmatrix} \cdot \begin{pmatrix} \boldsymbol{U} \\ \boldsymbol{\Omega} \end{pmatrix}, \tag{S1}$$

where the first matrix on the right side of the equation is called a 6×6 resistive matrix $\boldsymbol{\xi}$,

$$\boldsymbol{\xi} = \begin{pmatrix} \boldsymbol{\xi}_T & \boldsymbol{\xi}_{TR} \\ \boldsymbol{\xi}_{RT} & \boldsymbol{\xi}_R \end{pmatrix}, \tag{S2}$$

where $\boldsymbol{\xi}_{TR} = \boldsymbol{\xi}_{RT}^{\mathrm{T}}$.

The diffusion matrix $\boldsymbol{D}$ is then calculated by the Einstein relation as [3]

$$\boldsymbol{D} = k_B T \boldsymbol{\xi}^{-1}, \tag{S3}$$

where

$$\boldsymbol{D} = \begin{pmatrix} \boldsymbol{D}_T & \boldsymbol{D}_{TR} \\ \boldsymbol{D}_{RT} & \boldsymbol{D}_R \end{pmatrix}. \tag{S4}$$

Note that here $\boldsymbol{D}_{TR} = \boldsymbol{D}_{RT}^{\mathrm{T}}$ because $\boldsymbol{\xi}_{TR} = \boldsymbol{\xi}_{RT}^{\mathrm{T}}$, as also shown by Happel and Brenner [2]. Using the above terms, the local apparent diffusion coefficient along the x-axis at the radial position, $\beta \equiv r/R$, can be obtained as

$$D_x(\beta) = D_{T_{xx}} + \left(\xi_{TR_{xz}}/\xi_{T_{xx}}\right)^2 D_{R_{zz}}. \tag{S5}$$

B. Local diffusion coefficient

In Fig. S2, we show the simulated local diffusion coefficients of various λ $(= a/R)$ and L/R. At small λ, both open and closed channels behave qualitatively the same; the local diffusion coefficients are large when the particle is near the center and become gradually smaller near the wall. In contrast, at high λ, the trend is reversed for a closed channel; the local diffusion coefficients near the channel wall are larger than at the center. In the open channels (e.g., $L/R = 5, 10$), the diffusion coefficients decrease near the channel wall even at high λ.

C. Effective radius of the channel

The effective radius of the square cross-sectional channel was obtained as follows. First, normalized diffusion coefficients in closed cylindrical channels were simulated for various confinement ratios with a step size of $\Delta\lambda = 0.01$ (e.g., $\lambda = ..., 0.5, 0.51, 0.52, ...$). The values in between were interpolated linearly. Then, this data sets obtained by simulations and linear interpolation were fitted to the two experimental diffusion coefficients (356 nm & 505 nm) in the closed channels by the method of least squares. We obtained the effective channel diameter, $2R \approx 922\,\mathrm{nm}$, which yields the confinement ratios, $\lambda \approx 0.386, 0.548$, for $2a = 356, 505\,\mathrm{nm}$, respectively. We used these confinement ratios for the simulations in Fig. 2.

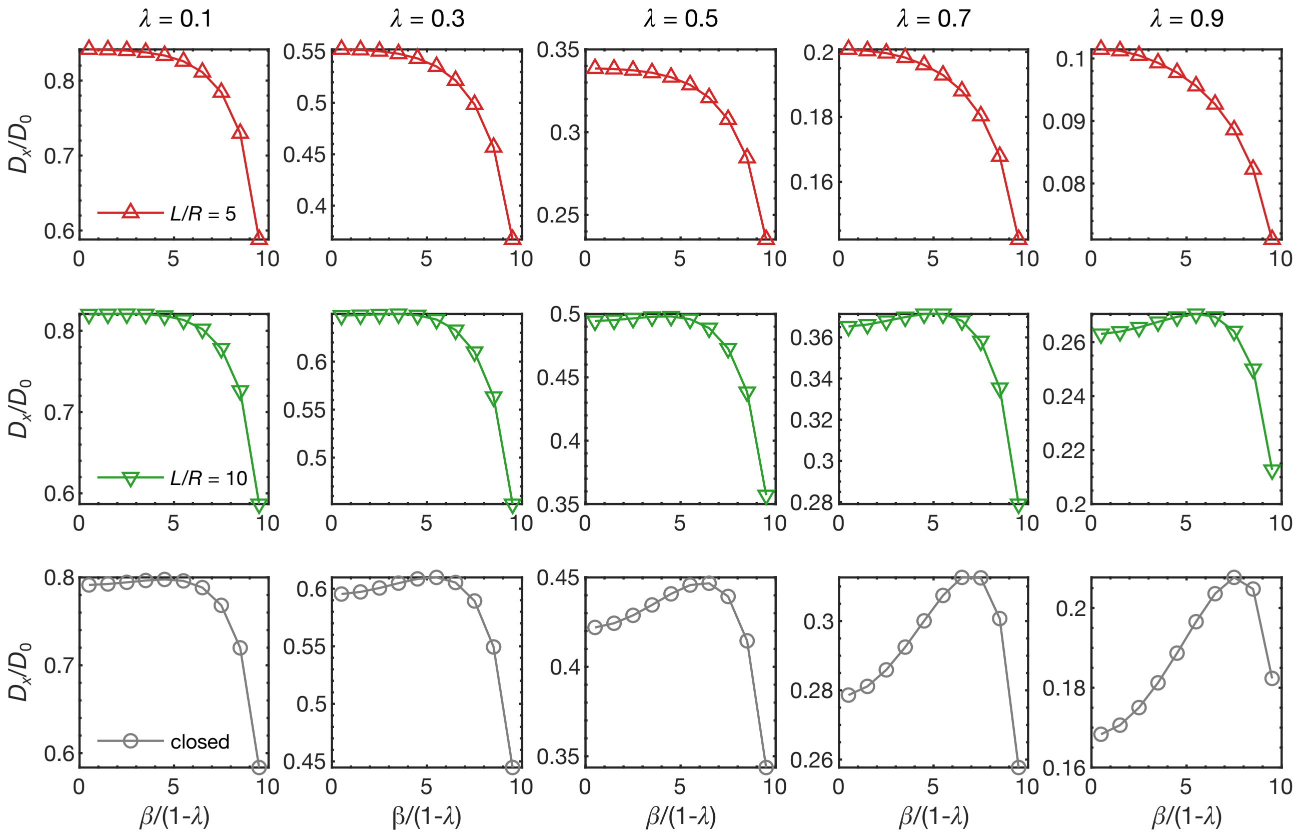

FIG. S2: Simulated local normalized diffusion coefficients of various confinement ratios λ and channel lengths as a function of the normalized radial position, $\beta/(1-\lambda)$, where $\beta = r/R$ and $\lambda = a/R$, respectively.

III. ANALYTICAL MODELING OF DIFFUSION COEFFICIENTS

A. Analytical expressions of matrix elements in Eq. 2

The matrix elements in Eq. 2 derived by Bungay and Brenner [4] are summarized in the following. The first and third rows of the matrix are

$$\begin{pmatrix} K_t \\ K_s \\ M_t \\ M_s \end{pmatrix} = \frac{9\pi\sqrt{2}}{4\,(1-\lambda)^{\frac{5}{2}}}\left[1+\sum_{n=0}^{2}\begin{pmatrix} a_n \\ b_n \\ c_n \\ d_n \end{pmatrix}(1-\lambda)^n\right]+\sum_{n=0}^{4}\begin{pmatrix} a_{n+3} \\ b_{n+3} \\ c_{n+3} \\ d_{n+3} \end{pmatrix}\lambda^n, \tag{S6}$$

where a_n, b_n, c_n, and d_n are, $a_1 = -73/60$, $a_2 = 77293/50400$, $a_3 = -22.5083$, $a_4 = -5.6117$, $a_5 = -0.3363$, $a_6 = -1.216$, $a_7 = 1.647$, $b_1 = 7/60$, $b_2 = 2227/50400$, $b_3 = 4.0180$, $b_4 = -3.9788$, $b_5 = -1.9215$, $b_6 = 4.392$, $b_7 = 5.006$, $c_n = b_n$ as $M_t = K_s$, and $d_1 = 29/20$, $d_2 = 97453/50400$, $d_3 = -62.2680$, $d_4 = -18.509$, $d_5 = -12.156$, $d_6 = 9.15$, $d_7 = 43.17$.

B. Compressibility of water

Here, we describe the empirical model of isothermal compressibility of water proposed by Fine & Millero [5]. The specific volume of water (the inverse of density, ρ^{-1}), $V(p,T)$, is modeled empirically as

$$V(p,T) = V_0 - \frac{V_0 p}{B + A_1 p + A_2 p^2}, \tag{S7}$$

where $A_{1,2}$ and B are temperature-dependent coefficients defined as

$$B = \left(19654.320 + 147.037T - 2.21554T^2 + 1.0478 \times 10^{-2}T^3 - 2.2789 \times 10^{-5}T^4\right) \times 10^5, \tag{S8a}$$

$$A_1 = 3.2891 - 2.3910 \times 10^{-3}T + 2.8446 \times 10^{-4}T^2 - 2.8200 \times 10^{-6}T^3 + 8.477 \times 10^{-9}T^4, \tag{S8b}$$

$$A_2 = \left(6.245 \times 10^{-5} - 3.913 \times 10^{-6}T - 3.499 \times 10^{-8}T^2 + 7.942 \times 10^{-10}T^3 - 3.299 \times 10^{-12}T^4\right) \times 10^{-5}. \tag{S8c}$$

Note that p and T are defined as a gauge pressure ($\equiv$ 'absolute pressure' $-$ 'atmospheric pressure') in pascals and as a degree Celsius, while in the referred literature [5] the unit of p is [bar]. V_0 is the specific volume at $p = 0$ (at 1 atm) defined as

$$V_0 = \left(1 + 18.159\,725 \times 10^{-3}T\right) / \Big(0.9998396 + 18.224\,944 \times 10^{-3}T - 7.922\,210 \times 10^{-6}T^2 - 55.448\,46 \times 10^{-9}T^3 + 149.7562 \times 10^{-12}T^4 - 393.2952 \times 10^{-15}T^5\Big). \tag{S9}$$

Thus, for a small pressure p, Eq. S7 can be simplified to

$$V\left(p, T\right) = V_0 - \frac{V_0 p}{B}. \tag{S10}$$

B is approximately 2.1789×10^9 Pa at $T = 20\,°\mathrm{C}$.

Figure S3 shows the experimentally measured specific volumes [5], the empirical model (Eq. S7), and the simplified model for a small applied pressure (Eq. S10) as a function of pressure. As the pressure in our system is sufficiently small, we use the model of Eq. S10.

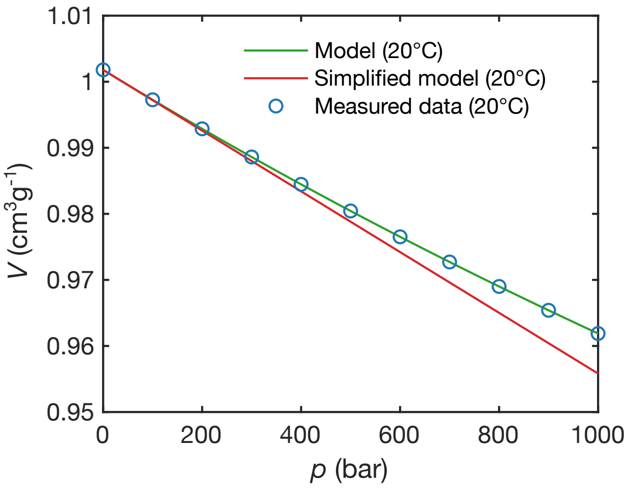

FIG. S3: Specific volume of water as a function of gauge pressure at 20 °C. Marker symbols indicate experimental data from [5].

C. Finite element simulation of a particle in a closed system

Here, we show time-dependent simulations of a particle in a compressible fluid under the influence of a constant force using the arbitrary Lagrangian-Eulerian method [6] to verify the analytical model shown in the main manuscript. In Fig. S4, the simulation results of normalized hydrodynamic frictions were plotted as a function of nondimensional time, t/τ. The simulation results showed excellent agreement with the analytical solutions.

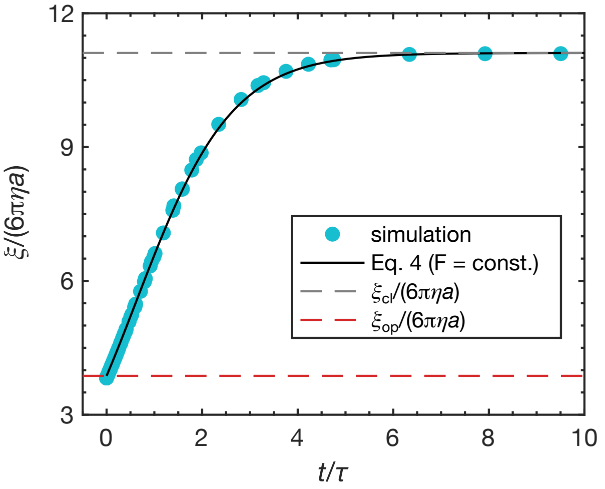

FIG. S4: Normalized hydrodynamic frictions of a particle translating under a constant force as a function of dimensionless time. The markers indicate the results of time-dependent simulations. The solid line indicates the analytical solution for a constant force condition. The top and bottom dashed lines indicate the analytical solutions of $\xi_{\rm cl}/(6\pi\eta a)$ and $\xi_{\rm op}/(6\pi\eta a)$, respectively. The geometric parameters used for the simulations are $a/R = 0.6$, $L/R = 5$, and $R_r/R = 10^3 \sim 10^4$ with a step size of 10^3.

[1] R. Dahiya, G. Gottardi, and N. Laidani, Microelectron. Eng. **136**, 57 (2015).
[2] J. Happel and H. Brenner, *Low Reynolds number hydrodynamics: with special applications to particulate media*, Vol. 1 (Springer Science & Business Media, 1983).
[3] D. W. Condiff and J. S. Dahler, J. Chem. Phys. **44**, 3988 (1966).
[4] P. M. Bungay and H. Brenner, Int. J. Multiph. Flow **1**, 25 (1973).
[5] R. A. Fine and F. J. Millero, J. Chem. Phys. **59**, 5529 (1973).
[6] S. Qian and Y. Ai, *Electrokinetic particle transport in micro-/nanofluidics: direct numerical simulation analysis* (CRC Press, 2012).
